# Arabinogalactan as Active Compound in the Management of Corneal Wounds: In Vitro Toxicity and In Vivo Investigations on Rabbits

**DOI:** 10.3109/02713683.2010.523193

**Published:** 2010-12-21

**Authors:** Susi Burgalassi, Nadia Nicosia, Daniela Monti, Giulia Falcone, Enrico Boldrini, Ortenzio Fabiani, Carla Lenzi, Andrea Pirone, Patrizia Chetoni

**Affiliations:** 1 Department of Pharmaceutical Sciences, University of Pisa, Pisa, Italy; 2 Opocrin SpA, Carlo di Formigine (MO), Italy; 3 Department of Animal Productions, Section of Anatomy, University of Pisa, Pisa, Italy

**Keywords:** Arabinogalactan, Cell cultures, Corneal wound healing, Histology, Rabbits

## Abstract

*Purpose:* Aims of the present investigation were to prove that natural polysaccharide arabinogalactan (AG) is well tolerated after ocular administration and exerts a high restoring effect on corneal epithelium abrasions.

*Materials and Methods:* AG interactions with corneal cells, as well as its effect on their proliferation, were evaluated employing rabbit corneal epithelial cell cultures. The effects due to the presence of benzalkonium chloride (BAK) were also studied on cell cultures, ex vivo on rabbit isolated corneas, evaluating the hydration level, and on the healing rate of experimental corneal wounds in rabbits. Furthermore, the healing process of corneal lesions treated with an experimental 5.0% AG solution was studied and compared with those obtained applying solutions of hyaluronic acid and tamarind seed polysaccharide, both chosen as a reference by virtue of their well-known adjuvant properties on corneal trophism; the study was carried out by light and transmission electron microscopy.

*Results:* BAK showed toxic effects on corneal epithelium in all experiments. AG proved to stimulate the growth of the corneal epithelial cells by interacting at the level of the cell plasma membrane. The microscopy observations of the epithelial surface of AG-treated damaged corneas revealed a well-restored and histologically organized ultrastrucrure characterized by fully formed microvilli and glycocalyx; the healing process resulted faster with respect to spontaneously recovered untreated corneas.

*Conclusion:* Our results suggest that AG can interact with corneal epithelial cells without any toxic side effect; moreover, it proved to stimulate cell proliferation, thus promoting tissue re-epithelial-ization and reorganization just 48 hr post-wounding.

## INTRODUCTION

The corneal epithelium is the first line of defense for the visual system and, like a barrier, is continuously subjected to physical, chemical, and biological insults, often resulting in wounds.^1^ Corneal abrasions, besides causing pain, tearing, photophobia and foreign body sensation, may rapidly progress to corneal scarring or even perforation with loss of barrier functions and subsequent invasion by pathogens. Proper healing of corneal wounds is essential for maintaining a clear, healthy cornea and for preserving vision.^2^ Corneal epithelium responds rapidly to injury and most corneal abrasions heal in 48–72 hr and rarely progress to corneal erosion.^3^ However, in contact lens wearers there may be rapid degeneration of corneal wounds.^4^ Due to this, during contact lens wearing, the administration of specific products able to prevent or to repair incidental corneal damages as soon as possible, can be useful.

The continuous search for compounds favoring cell adhesion and promoting ocular wound healing prompted us to investigate the utility of arabinogalactan (AG), a natural polysaccharide from the Larch tree, in the repair of corneal wounds.

In a previous study AG solution showed mucoadhesive properties that could be promising in terms of retention on the eye surface. Additionally, such formulations seemed to avoid/reduce the onset of dry spots on the corneal epithelium; finally, they significantly increased the healing rate of corneal wounds, with respect to other polymers commonly used as adjuvants in ophthalmic vehicles.^5^

Therefore, the present study was aimed at investigating AG ocular tolerance, its ability to interact with corneal cells, and its restoring action on corneal epithelium abrasions. Histological evidence of the healing process improvement of corneal wounds after AG treatment was found and discussed.

## MATERIALS AND METHODS

### Products

Arabinogalactan, AG (OPA 3816, Opocrin SpA, Corlo di Formigine, Italy); mannitol and benzalkonium chloride, BAK (Carlo Erba, Milan, Italy); hyaluronic acid, HA (Chemofin, Milan, Italy); tamarind seed polysaccharide, TSP (TSP® eyedrops, Farmigea S.p.A., Pisa, Italy); cell proliferation reagent WST-1 (Roche Diagnostics GmbH, Mannheim, Germany); isothiocya-natofluorescein, dibutyltin dilaurate, and 1-heptanol (Sigma-Aldrich, Milan, Italy).

The rabbit corneal epithelial cell line (RCE) was obtained from the European Cell Culture Collection (no. 95081046, ECACC, Salisbury, Great Britain). The growth medium had the following composition: DMEM/F-12 (1:1) supplemented with fetal bovine serum (15% v/v), L-glutamine (1% v/v, 2mM), penicillin (100 Ul/ml), streptomycin (O.lmg/ml), and amphotericin B (0.25 ng/ml) (Invitrogen, Milan, Italy), insulin (5 (xg/ml), and epidermal growth factor (lOng/ml) (Sigma-Aldrich®, Milan, Italy).

All other chemicals, solvents, etc. were of analytical grade.

### Animals

Female albino New Zealand rabbits (Pampaloni Rabbitry, Fauglia, Italy), weighing 2.5–3.0 Kg were used and treated under veterinary supervision according to the “ARVO Statement for the Use of Animals in Ophthalmic and Vision Research”; the experimental protocols were approved by the Ethical-Scientific Committee of the University of Pisa.

Animals were housed in standard single cages under controlled lighting, at 19 ± 1°C and 50 ± 5% R.H., without any restriction of food or water. During the experiments, rabbits were placed in restraining boxes, to which they had been habituated, and kept at lower lighting; heads were allowed to move freely without any restriction of eye movements.

### Test Formulations

#### Formulations for Toxicity Tests

The test substances were AG and BAK. For cytotoxicity studies, solutions of AG and AG/BAK were prepared in DMEM.

For corneal hydration tests, solutions containing 2.0% w/w AG or 0.004% w/w BAK or a combination of 2.0% w/w AG and 0.004% w/w BAK in pH 6.85 glutathione bicarbonate Ringer buffer (GBR) were employed.

#### Formulations for In Vivo Tests

A 5.0% w/w AG solution (AG-Sol), a 0.2% w/w HA solution (HA-Sol), and a 5.0% w/w AG added of 0.01% w/w BAK solution (AG-BAK) were prepared in pH 7.4, 2.66mM phosphate buffer solution (PBS), containing 4.0% w/w mannitol for isotonicity. All formulations were sterilized by filtration through cellulose acetate membrane (0.22 µm), stored in sealed vials, and used immediately after opening.

0.5% TSP® commercial eyedrops, containing tamarind seed polysaccharide and mannitol for isotonicity, were used.

### Synthesis and Characterization of Fluorescein-Labeled AG

Fluorescein-labeled arabinogalactan (FITC-AG) was synthesised following De Belder's method:^6^ arabinogalactan (1 g) was dissolved in methyl sul-phoxide (10 ml) containing a few drops of pyridine. Isothiocyanatofluorescein (0.1 g) was added, followed by dibutyltin dilaurate (20 mg), and the mixture was heated for 2hr at 95°C in water bath. After several precipitations in ethanol to remove free dye, the FITC-AG was filtered off and dried at 80°C.

FITC-AG aqueous solutions were prepared by gentle stirring and heating for 5min at 80°C; the degree of substitution (d.s.) was determined by fluorospectrometry (Shimadzu RF-551 equipment; excitation and emission wavelengths 490 and 514 nm, respectively, Shimadzu Corporation, Kyoto, Japan) and calculated as molar ratio of fluorescein to the repeating unit of AG, considering galactose and arabinose units in a molar ratio of 8:1 (MW weighted mean 1168Da).^7^

### Studies on Corneal Epithelial Cell Cultures

Immortalized rabbit corneal epithelial (RCE) cells were chosen as the model culture; the cells were grown at 37°C in a humidified atmosphere containing 5% CO_2_. The cell viability test was based on the ready-to-use cell proliferation reagent WST-1.

#### Cytotoxicity Test

RCE cells, passage numbers 20–23, were plated at a density of 5 × 10^3^ cells/well in a 96-well microtiter plate (Corning Costar®, Milan, Italy). Twenty-four hours after plating, at 70% confluence and before the cultures became multilayered, the growth medium was removed and replaced with the test solutions (100 µl). After a predetermined time of exposure at 37°C in humidified atmosphere with 5% CO_2_, the reaction medium was discarded, the cells were washed twice with pH 7.4 PBS, and fresh growth medium (100 µl) and cell proliferation reagent WST-1 (10 µl) were added in each well. The cells were again incubated in humidified atmosphere at 37°C and 5% CO_2_ for 2hr, then the multititer plate was thoroughly shaken for 9 sec and absorbances were read at 450 nm using a microtiter reader (Microtiter reader 550®, Bio-Rad Laboratories, Hercules, California, USA). The use of a 2-hr incubation period was based on a series of preliminary experiments. The background absorbance was measured on wells containing only the dye solution and the culture medium.

The results were expressed as percent optical density of treated vs. control untreated wells.

Cell viability was tested after 1 hr exposure at different concentrations of AG-BAK and after 1, 24,48, and 72 hr exposure at AG.

#### AG Cell Interaction

To evaluate the interactions between AG and corneal epithelial cells, RCE cells, passage numbers 20–22, were seeded in six-well culture slides (500 cells/well) and incubated in humidified atmosphere at 37°C and 5% CO_2_ in the presence of growth medium; after 24 hr wells were treated or not (control) with FITC-AG solution (final concentration in well 2.5 and 5.0mg/ml) and incubated for 60min; the cells were then handled for microscopic examination as described afterwards in the “Microscopy Techniques” section.

### Evaluation of Corneal Hydration Levels

To investigate the corneal hydration induced by AG, a gravimetric method involving desiccation of the tissue was used. The animals were euthanized with an intravenous lethal dose of sodium pentobarbital (Pentothal sodium, Farmaceutici Gellini, Aprilia, Italy). The eyes were then proptosed and the corneas, with a 2-mm ring of sclera, were immediately excised and mounted in perfusion cells^8^ maintained at 32±0.5°C. The cells, made of acrylic plastic, consisted of a donor compartment (epithelial side, volume 1.0 ml) and a receiving compartment (endothelial side, volume 5.0ml). The area occupied by the cornea between the two compartments was 0.78 cm^2^. After positioning the cornea in the apparatus, 5.0 and 1.0 ml of preheated pH 6.85 GBR, were added to the receiving and donor compartments, respectively. To assure oxygenation and agitation, a mixture of 95% O_2_–5% CO_2_ was bubbled through each compartment at rate of 3–4 bubbles/sec. After allowing the corneal conditions to equilibrate for 10 min, the solution on the epithelial side was withdrawn and substituted with 1.0 ml of a solution of an agent under study and kept in contact with the tissue for 1.0 hr. Each experiment was repeated 12 times.

At the end of the experiment, the cornea was removed from the perfusion apparatus, any surface water was removed by gently blotting with filter paper, and the scleral ring was removed too; the corneal sample was weighed and desiccated at 100°C to a constant weight (about 12hr). The percent of corneal hydration level (HL%) was obtained as follows:


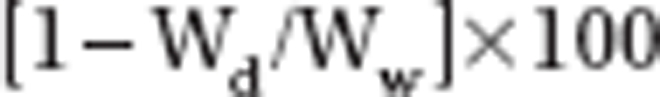


where W_d_ and W_w_ are the dry and wet corneal weights, respectively.

### In Vivo Studies: Experimental Corneal Lesions

Corneal surfaces were preliminarily examined by a slit-lamp and 53 rabbits, with healthy corneal epithelium, were selected and divided into six groups. Fifty animals underwent experimental corneal lesions, as described in a previous paper.^9^ Briefly, animals were anesthetized; the right eye was then kept open by a blepharostat and anesthetized in surface by 10 µl of oxybuprocaine hydrochloride; the corneal epithelium was removed by applying a paper disc for (diameter 6 mm) soaked with 10 µl of n-heptanol 1 min; finally, the eyes were carefully rinsed with normal saline. These animals received 50 µl of test formulation or vehicle (PBS, control groups), three times daily in the damaged eyes only, while the eyes of three animals were kept healthy and treated three times daily with 50 µl of test formulation. The groups were treated and evaluated following the scheme below:

- Groups 1, 2, and 3 (eight animals in each one) received AG-Sol, AG-BAK, or vehicle, respectively. Immediately after producing the epithelial damage and before each measurement, the right eye was stained with 10 µl of sodium fluorescein (1% w/w in water) to visualize the damaged area, and images were taken using a slit-lamp equipped with a blue filter and camera. Computer analysis of the images using ImageJ (public domain software, National Institute of Mental Health, Bethesda, Maryland, USA) was employed to determine the wound area at each time point.
- Group 4 (eight animals) was treated with AG-Sol, HA-Sol, TSP, or vehicle (two animals each treatment); animals were sacrificed immediately before complete recovery of the corneal lesion, when fluorescein captation on the ocular surface was still visible, but the lesion was no longer measurable; finally, the corneal buttons were excised and handled for light microscopy examination as described in the “Microscopy Techniques” section.
- Group 5 (eighteen animals) was treated with AG-Sol or vehicle (nine animals each treatment); animals were sacrificed at predetermined time intervals (24 hr, 48 hr, or 7 days after lesions), then corneal buttons were excised and bisected for light (semi-thin) and transmission electron microscopy as described in the “Microscopy Techniques” section.
- Group 6 (three animals with healthy corneas) was treated with AG-Sol for a month; animals were then sacrificed and the corneal buttons excised and treated as described above for group 5.

### Microscopy Techniques

#### Fluorescence Microscopy

The cultured cells were rinsed twice with PBS and fixed in 10% paraformaldehyde-PBS solution (0.1 M, pH 7.4), then rinsed with depurated water. Fluorescent specimens were analyzed using a Zeiss Axioskop microscope (Carl Zeiss GmbH, Jena, Germany), and digital images were taken with a Leica DClOO camera (Leica, Wetzlar, Germany).

Light microscopy (LM) was performed fixing samples in a 10% paraformaldehyde-PBS solution (0.1 M, pH 7.4); samples were washed with PBS, dehydrated in a graded ethanol series, then embedded at 4°C in a specific resin for light microscopy (JB-4, Embedding kit, Polysciences Inc., Warrington, Pennsylvania, USA). Finally, embedded samples were sectioned by microtomy, stained following Nissl's method or with methylene blue/toluidine blue, observed and photographed by a light microscope (Diaplan, Leitz, Stuttgart Baden-Wurttemberg, Germany).

For transmission electron microscopy (TEM) specimens were fixed in Karnowsky solution (2.5% glutar-aldehyde and 4.0% paraformaldehyde in 0.1 M, pH 7.4 PBS) for 3hr, washed in PBS, post-fixed in 1.0% osmium tetroxide in PBS, and dehydrated in a graded ethanol series; specimens were then washed with propylene oxide and embedded in epoxy resin media (Epon-Araldite, Fluka, Buchs, Switzerland). Ultra-thin sections of 60 nm were cut by a diamond knife on an ultra-microtome (LKB-Huxley, LKB Instruments Inc., Rockville, Maryland, USA). Sections were collected on copper grids, stained with uranyl acetate and lead citrate, then examined with a transmission electron microscope (Siemens 101, Siemens, Milan, Italy).

## RESULTS AND DISCUSSION

### AGToxicity Investigations

Experiments were aimed at investigating a possible AG ocular toxicity by evaluation of cell viability and corneal hydration levels.

**FIGURE 1:**
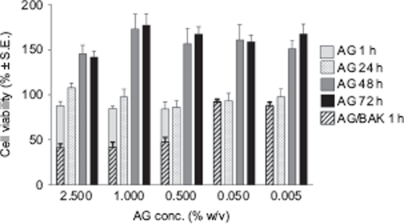
Cell viability over the time after RCE exposure to different concentrations of AG and AG/BAK vs. AG concentration.

Figure 1 illustrates the viability, as percentage survived with respect to the control, of cells exposed to various concentrations of AG and AG/BAK vs. AG concentration. It can be noted that AG did not show any cytotoxicity on RCE substrate; even better, at concentrations ranging from 5 × 10^−3^ to 2.5% w/v (from 50 to 25,000 µg/ml), it enhanced the growth of the corneal epithelial cell cultures: increasing the exposure time for up to 72 hr cell viability reached values even 1.8-fold higher with respect to the control, thus, indicating a stimulation of cell proliferation. Miyazaki and co-workers found a similar effect for sodium hyaluronate (NaHA): 500–1000 µg/ml of NaHA were able to increase the growth of RCE cell cultures up to about 1.5-fold. In our study, AG showed a similar promoting activity but at a significantly lower concentration (50 µg/ml).^10^

When BAK was added to AG solution (0.01%, concentration normally used as preservative in ophthalmic solution) the RCE cytotoxicity was considerably raised, producing cell viability values lower than 50% for the higher BAK concentrations, confirming its cytotoxic activity reported in scientific literature.^11^–^14^

The second series of experiments was carried out to highlight possible alteration of entire cornea after AG contact knowing that water content into the tissue (normally ranging from 76 to 80%) increases in case of damage of the structure.^15^,^16^ The percent corneal hydration values determined gravimetrically after 1 hr treatment are listed in Table 1. The HL% of corneas kept in contact with GBR alone was 78.80 ± 0.36%; this value, taken as a reference, was lower than that observed in a previous study by the same research group,^17^ but in agreement with previous literature data.^18^,^19^ As listed in the table, all treatments induced an increase of HL% of corneas even if the difference was statistically significant only for corneas treated with BAK alone, which HL% reached 79.98 ± 0.15%, again demonstrating the lack of toxicity of AG and the negative affects on the cornea due to the contact with benzalkonium chloride.

**TABLE 1:** Percent corneal hydration values determined gravimetrically after 1 hr of treatment

Formulation	HL%^a^
GBR	78.76 ± 0.36
AG 2.0%	79.57 ± 0.44
BAK 0.004%	79.98 ± 0.36*
AG 2%/BAK 0.004%	79.68 ±0.49

a Mean±SE,w = 12.

* Significantly different (*P*<0.05, *t*-test unpaired two-tailed) from GBR.

### AG Re-Epithelializing Activity

**FIGURE 2:**
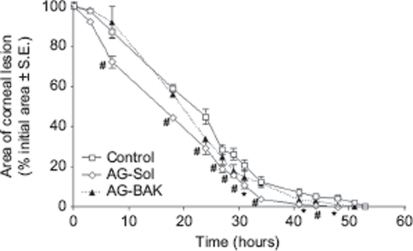
Reduction of the corneal defect area in rabbits after treatment with AG-Sol and AG-BAK solution (*n* =8). Significantly different (Student's unpaired *t*-test) from control, *P<0.05, #*P*<0.01 level.

Figure 2 graphically shows the corneal lesion healing trend over time for the animal groups 1–3. The data are reported as percentage of the area of the lesion, taking as 100% the area at time 0. AG-Sol formulation produced a small decrease in the damaged area since the first treatments with respect to AG-BAK and control groups. Moreover, as shown by statistical analysis, AG-Sol produced significantly different results from the control already 7hr after the first treatment, whereas no time points were significantly different for AG-BAK. The complete recovery in the animals treated with AG-BAK formulation occurred after 51 hr, happening shortly before the control group (53 hr), while in the animals treated with AG-Sol, it occurred as early as 44 hr after. In line with these observations and the previous results that demonstrated BAK corneal toxicity, the product containing AG with re-epithelializing activity, object of a patented invention, rejects the presence of BAK.^20^

**FIGURE 3:**
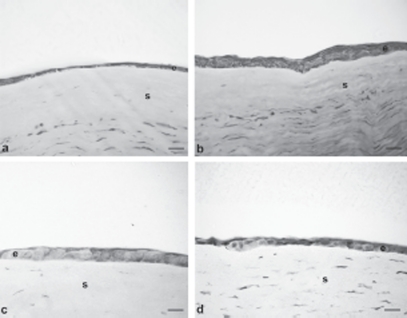
Photomicrographs of damaged corneas, recovered spontaneously [(A) control, thickness 6 µm], or treated with: AG-Sol [(B) thickness 18 µm]; TSP [(C) thickness 18 µm]; and HA-Sol [(D) thickness 12 µm]; corneas were excised immediately before complete recovery of the corneal lesion. Scale bar = 20 µm. Legend: e = epithelium; s = stroma.

In our previous article, the treatment of damaged corneas with different polysaccharides (hyaluronic acid and tamarind seed polysaccharide) highlighted a decrement in the healing time that strictly depended on the product applied, thus suggesting an influence of polymers on the restoration mechanism.^5^ Therefore, the re-epithelialization process was deeply investigated and characterized in order to explain these findings. Figure 3 represent histological images obtained from treated corneas and untreated cornea reference, all excised immediately before complete recovery of the corneal lesion (animal group 4); for each sample the relevant thickness of the reconstituted epithelium, measured at constant distance from the re-growth margin, is reported. Corneal samples resulted different in morphology and thickness: samples deriving from control eyes showed a quite poorly stratified and unorganized structure; thickness was still much lower than in the native cornea (6 µm vs. 45–47 µm in the central region). AG-Sol-treated epithelium showed a close similarity to native epithelium in terms of tissue arrangement, even if thickness was still reduced (18 µm). TSP-treated sample revealed a poorly stratified neo-tissue, even if no significant difference in thickness, with respect to AG-Sol, was evidenced. An explanation of this result could be tentatively attributed to the presence of a few cells much larger than normal. Treatment with HA determined a discrete stratification of the neo-epithelium, even if the final thickness obtained was still low (12 µm).

In the early hours after a corneal injury occurred, the physiological reparative process leads to formation of a cell monolayer by shift, adhesion to the extracellular matrix and migration of the adjacent epithelial cells to the damaged area. All these events are mediated by integrins, adhesion receptors connecting extracellular matrix to cytoplasm elements;^21^ since polysaccharides can influence integrins recognition,^22^ they reasonably influence cell adhesion and, as a consequence, the whole healing process. This activity, combined with the higher replication ability that epithelial cells showed in the presence of AG would lead to a faster stratification of the tissue. An interaction of the product with cell surface was suggested by the results obtained after exposure of RCE to fluorescein-labeled AG.

**FIGURE 4:**
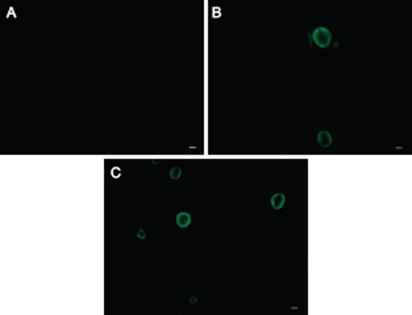
Fluorescence photomicrographs of rabbit corneal epithelial cells. (A) cells untreated; (B) and (C): cells incubated for 60min with 2.5 and 5.0mg/ml AG-FITC derivative, respectively. Scale bar, 10 µm.

Digital images of cells kept in contact with FITC-AG are shown in Figure 4. RCE cells did not show basal fluorescence, as demonstrates the image of untreated cells (4A), whereas a green fluorescence extensively localized on plasma membranes was evident when the cells were incubated both with 5.0 and 2.5mg/ml AG-FITC (d.s. 0.02–0.005) solution.

An in-depth histological evaluation of AG treated corneas was carried out to assess whether the re-epithelialization process—besides providing for the complete healing of the lesions—also determined the reconstitution of the original corneal structure.

**FIGURE 5:**
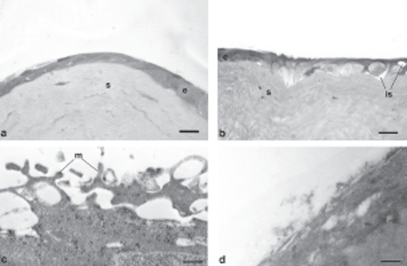
Photomicrographs of corneal epithelium structure at LM [(A) AG-Sol treated, and (B) control; scale bar = 10 µm] and at TEM [(C), AG-Sol treated, and (D) control; scale bar=1 µm) 24 hr post wounding. Legend: e = epithelium; is = intercellular space; m=microvilli; s = stroma.

**FIGURE 6:**
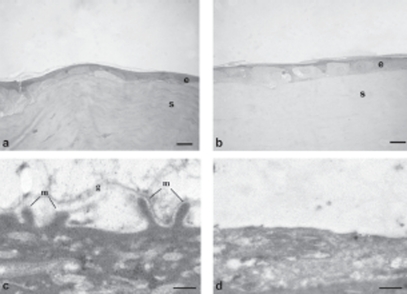
Photomicrographs of corneal epithelium structure at LM [(A) AG-Sol treated, and (B) control; scale bar = 10 µm] and at TEM [(C) AG-Sol treated, and (D) control; scale bar = 1 µm] 48 hr post wounding. Legend: e = epithelium; g = glycocalyx; m = microvilli; s = stroma.

**FIGURE 7:**
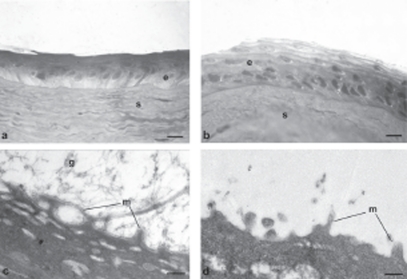
Photomicrographs of corneal epithelium structure at LM [(A) AG-Sol treated, and (B) control; scale bar = 10 µm] and at TEM [(C) AG-Sol treated, and (D) control; scale bar = 1 µm) 7 days post wounding. Legend: e = epithelium; g = glycocalyx; m = microvilli; s = stroma.

**FIGURE 8:**
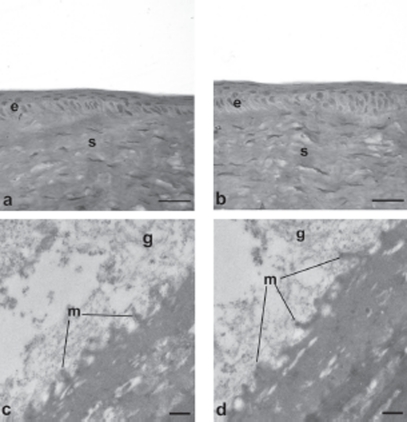
Morphological features of a healthy cornea at LM [(A) AG-Sol treated, and (B) control; scale bar = 10 µm] and at TEM [(C) AG-Sol treated, and (D) control; scale bar = 1 µm). Legend: e = epithelium; g = glycocalyx; m = microvilli; s = stroma.

Figures 5–8 are images obtained by light and transmission electron microscopy (LM and TEM, respectively) conducted at different time points on animal group 5; such techniques represented suitable methods to observe the migration of cells surrounding the wounded area after treatment with AG-Sol or vehicle.

The epithelium of AG-treated corneas since 24 hr post wounding displayed a continuous organized structure with the presence of microvilli, while control epithelium was characterized by cells of different sizes and intercellular spaces. The treated epithelium develops into a natural structure (presence of microvilli and glycocalyx materials) in 48 hr post wounding, while the control still showed an unorganized structure characterized by larger than normal cells. Several days after complete healing, 7 days post wounding, the superficial epithelium of control corneas still showed no well histologically organized epithelium with cells not fully differentiated and absence of glycocalyx.

Comparing healthy corneas treated with AG-Sol for 30 days (animal group 6) and native tissue, no morphological differences were noticed, indicating that AG doesn't stimulate proliferation of intact epithelium even though its proliferative activity on RCE has been demonstrated. This data should exclude any toxic effect of AG towards healthy epithelium.

## CONCLUSIONS

The results of this study suggest the potential of AG, possibly as a single-dose formulation or in the presence f a preservative different from benzalkonium, in the management of corneal wounds. AG solutions might particularly benefit contact-lens wearers, on account of their tolerability prolonged permanence, and noninterference with vision, due to low viscosity.

Further studies meant to verify the complete compatibility of AG with different types of contact lens and to investigate the biological mechanisms involved in the healing process enhanced by AG are in progress.
